# Prolonged nucleic acid conversion and false-negative RT-PCR results in patients with COVID-19: A case series

**DOI:** 10.1016/j.amsu.2020.09.040

**Published:** 2020-10-06

**Authors:** Ika Trisnawati, Riat El Khair, Dyah Ayu Puspitarani, Aditya Rifqi Fauzi, Gunadi

**Affiliations:** a Pulmonology Division, Department of Internal Medicine, Faculty of Medicine, Public Health and Nursing, Universitas Gadjah Mada/Dr. Sardjito Hospital, Yogyakarta, 55281, Indonesia; b Department of Clinical Pathology and Laboratory Medicine, Faculty of Medicine, Public Health and Nursing, Universitas Gadjah Mada/Dr. Sardjito Hospital, Yogyakarta, 55281, Indonesia; c Genetics Working Group, Faculty of Medicine, Public Health and Nursing, Universitas Gadjah Mada/Dr. Sardjito Hospital, Yogyakarta, 55281, Indonesia

**Keywords:** COVID-19, Case series, False negative of RT-PCR, Prolonged nucleic acid conversion, SARS-Cov-2

## Abstract

**Background:**

Prolonged nucleic acid conversion and false-negative real-time polymerase chain reaction (RT-PCR) results might occur in COVID-19 patients rather than infection recurrence.

**Presentation of cases:**

We reported four cases who had negative RT-PCR results, in addition to the last two consecutive negative results. Patient-1 had negative RT-PCR results twice (the 6th and 8th) from a total of 11 swabs. Patient-2 had negative RT-PCR results once (the 5th) from a total of 8 swabs. Patient-3 showed negative results of RT-PCR twice (the 4th and 6th) from a total of 11 swabs. Patient-4 had negative RT-PCR results twice (the 2nd and 10th) from a total of 14 swabs.

**Discussion:**

The fluctuating trend of our RT-PCR results in our cases might be due to insufficient viral material in the specimen, laboratory errors during sampling, restrictions on sample transportation, or mutations in the primary and probe target regions in the SARS-CoV-2 genome. Several factors might affect the occurrence of prolonged nucleic acid conversion, including older age, comorbidities, such as diabetes and hypertension, and impaired immune function.

**Conclusion:**

Here, we confirmed the occurrence of prolonged nucleic acid conversion and the possibility of false negative RT-PCR results in COVID-19 patients.

## Highlights

• Prolonged nucleic acid conversion in patients with COVID-19 from Indonesia, becoming the longest reported of 70 days.
• We further confirmed the occurrence of prolonged nucleic acid conversion and false negative RT-PCR in COVID-19 patients.
• Several factors might affect the prolonged nucleic acid conversion, including older age and comorbidities.

## 1 Introduction

In December 2019, an outbreak of infection of severe acute respiratory syndrome-coronavirus-2 (SARS-CoV-2) was detected in Wuhan, China. This virus has the etiology of Coronavirus disease 2019 (COVID-19), first announced by the World Health Organization (WHO) on January 12, 2020, and now becomes a global pandemic [1,2].

The length of the virus incubation period is approximately five days [3]. Several studies [4,5] reported a median length of viral shedding between 12 and 20 days from the onset of symptoms. Previously, cases of prolonged nucleic acid conversion have also been reported, with the longest reported being 60 days [6]. Nucleic acid conversion is defined as the period from the date of symptom onset to the date of the first negative real-time reverse transcription polymerase chain reaction (RT-PCR) test result [7]. It has been hypothesized that prolonged nucleic acid conversion and false negative results of RT-PCR occur in some patients with COVID-19 rather than recurrence of infection [7]. Here, we report four cases of COVID-19 with the possibility of prolonged nucleic acid conversion and false negative results of RT-PCR in our institution, Indonesia. This case series has been reported in line with the PROCESS criteria [8].

## 2 Presentation of cases

### 2.1 Case 1

**Fig. 1:**
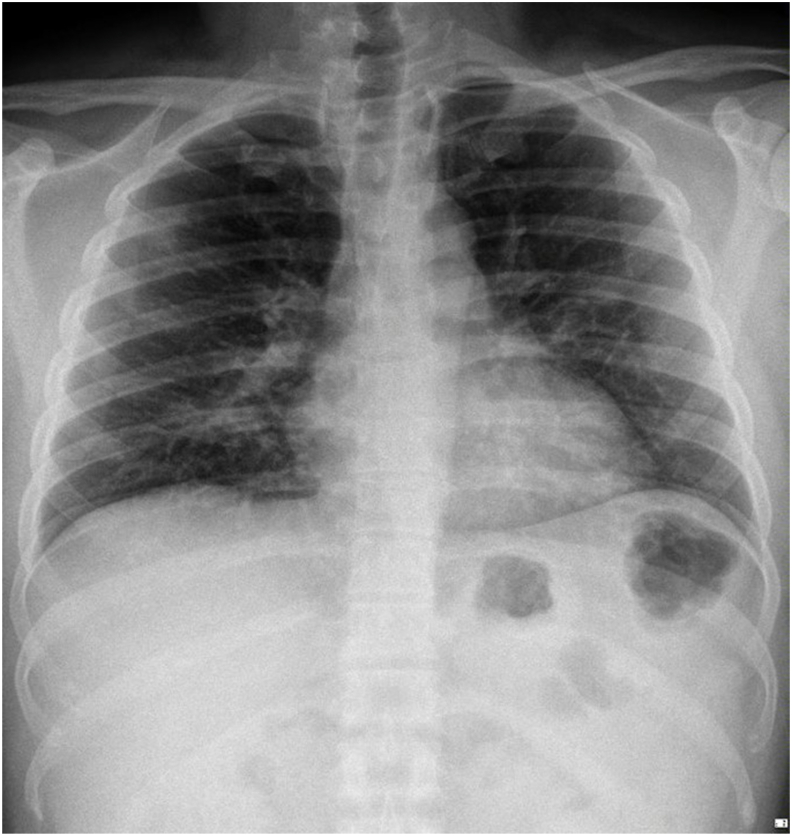
Chest X-ray indicated mild pneumonia in the right lung.

A 36-year-old male patient complained of coughing up phlegm for one week before admission. He had a history of contact with a positive confirmed case of COVID-19 on March 13, 2020, and swab tests were conducted on March 24 and 25, 2020, with positive results. The physical examination recorded a blood pressure of 187/94 mmHg, with normal results on his remaining vital signs. Lung auscultation revealed no apparent abnormality. Chest X-rays showed the appearance of mild pneumonia in the right lung (Fig. 1). Blood tests showed an increase in the neutrophil-lymphocyte ratio (NLR) of 2.03 and *C*-reactive protein (CRP) of 10 mg/L. After admission, the patient received antibiotics and antiviral therapy based on the COVID-19 Prevention and Control guidelines by the Indonesian Ministry of Health, namely, azithromycin, hydroxychloroquine, oseltamivir, lopinavir-ritonavir, and umifenovir. SARS-CoV-2 retesting with nasopharyngeal and oropharyngeal swabs was performed with positive results in the 3rd until 5th test. In the 6th swab, the results were negative but were positive in the 7th swab. In the 8th swab, the results returned negative but were positive in the 9th swab. Two consecutive negative results were found on the 10th and 11th swabs. The patient was discharged from the hospital on April 25, 2020, 31 days after the onset of illness (Table 1).

**Table 1:** RT-PCR findings in COVID-19 patients treated in Dr. Sardjito Hospital, Indonesia.

Patients	Age	Gender	RT-PCR 1	Test days, after onset	RT-PCR 2	Test days, after onset	RT-PCR 3	Test days, after onset	RT-PCR 4	Test days, after onset	RT-PCR 5	Test days, after onset	RT-PCR 6	Test days, after onset	RT-PCR 7	Test days, after onset	RT-PCR 8	Test days, after onset	RT-PCR 9	Test days, after onset	RT-PCR 10	Test days, after onset	RT-PCR 11	Test days, after onset	RT-PCR 12	Test days, after onset	RT-PCR 13	Test days, after onset	RT-PCR 14	Test days, after onset
Case 1	36	Male	+	1	+	2	+	8	+	10	+	14	-	16	+	19	-	21	+	23	-	28	-	31	N/A		N/A		N/A	
Case 2	54	Male	+	2	+	6	+	10	+	14	-	18	+	20	-	24	-	29	N/A		N/A		N/A		N/A		N/A		N/A	
Case 3	47	Male	+	10	+	15	+	22	-	28	+	32	-	37	+	40	+	44	-	50	-	52	N/A		N/A		N/A		N/A	
Case 4	56	Female	+	15	-	16	+	22	+	27	+	32	+	37	+	42	+	48	+	53	-	58	+	61	+	64	-	68	-	70

### 2.2 Case 2

**Fig. 2:**
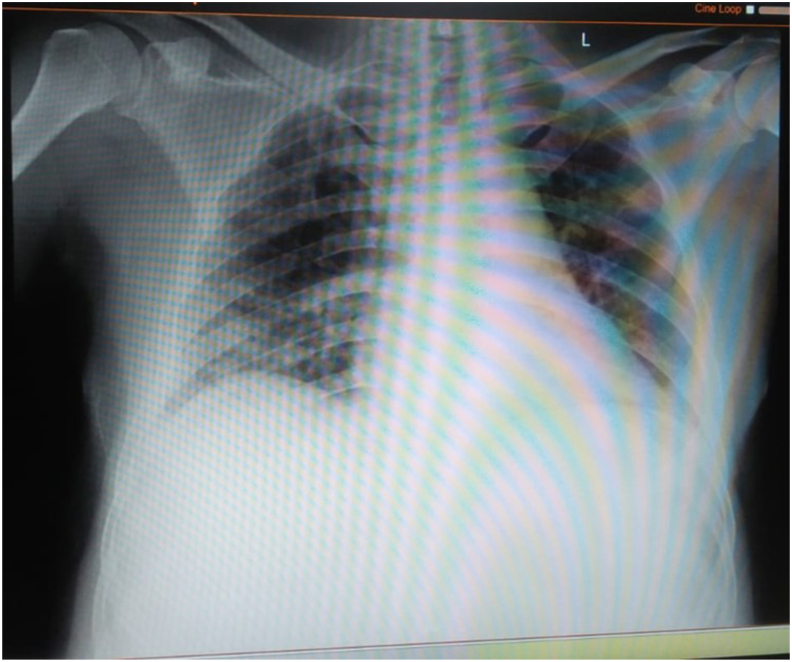
Chest X-ray revealed inhomogeneous opacity on bilateral paracardial and lateral aspects.

A 54-year-old male patient complained of shortness of breath that worsened with activity. The patient began experiencing fever nine days before admission. Three days before admission, he experienced a cough with phlegm. He had a history of contact with a confirmed case of COVID-19 two weeks before admission. He had a comorbidity of diabetes controlled with routine medication. The physical examination recorded blood pressure of 131/72 mmHg, pulse of 96 per minute, respiratory rate of 24 per minute, body temperature of 38 °C, and oxygen saturation of 97% with oxygenation of 2 L per minute using nasal cannula. Lung auscultation revealed no apparent abnormality. Rapid diagnostic tests using SARS-CoV-2 antibody were performed and showed non-reactive results. Chest X-rays showed inhomogeneous opacity on bilateral paracardial and lateral aspects, typical of viral pneumonia caused by COVID-19 infection (Fig. 2). We found increases in the NLR and CRP of 2.94 and 107 mg/L, respectively. After admission, the patient received antibiotics and antiviral therapy based on the COVID-19 Prevention and Control guidelines by the Indonesian Ministry of Health, namely, azithromycin, hydroxychloroquine, and lopinavir-ritonavir. On the following day, naso/oropharyngeal swabs were performed with positive results. On the fifth day of treatment, naso/oropharyngeal swabs were performed again, and the results were still positive, even though his symptoms were relieved. Positive PCR results were found until the 4th test but were negative on the 5th test, turned positive again on the 6th, and then two consecutive results were found to be negative on the 7th and 8th tests (Table 1). The patient was uneventfully discharged after 29 days of treatment.

### 2.3 Case 3

**Fig. 3:**
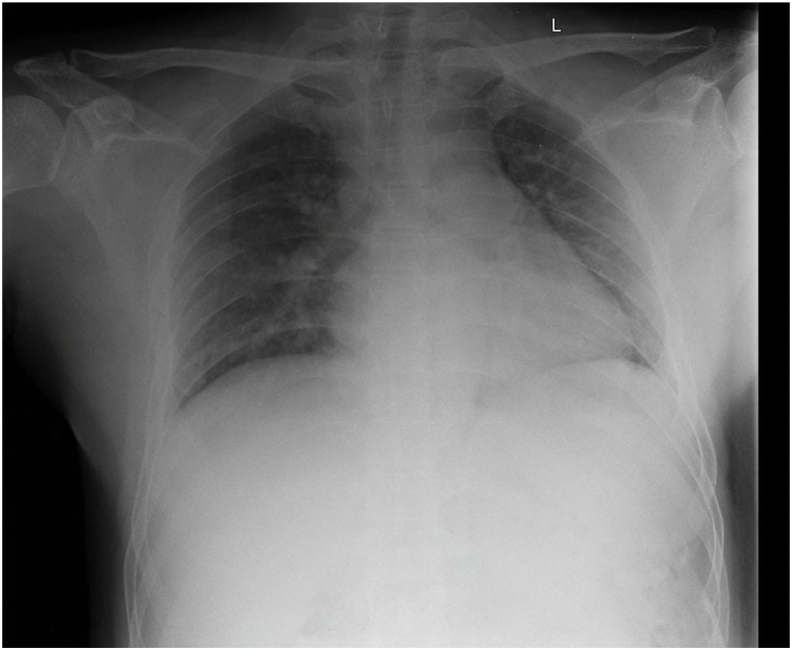
Chest X-ray revealed bilateral pneumonia.

A 47-year-old man presented with complaints of fever, cough, sore throat, and diarrhea that were experienced from 10 days before admission. The patient has comorbidities of asthma and heart rhythm disorders in the form of ventricular extra systole. He had a history of penicillin allergy. His vital signs are within normal limits. Lung auscultation revealed crackles in both lungs. Rapid diagnostic tests using SARS-CoV-2 antibodies were performed and showed reactive results. Chest X-rays showed bilateral pneumonia (Fig. 3). We found increases in the NLR and CRP of 20.90 and 32 mg/L, respectively. A blood culture test was performed and showed negative bacterial growth. After admission, the patient received antibiotics and antiviral therapy based on the COVID-19 Prevention and Control guidelines by the Indonesian Ministry of Health, namely, azithromycin, hydroxychloroquine, umifenovir and lopinavir-ritonavir. On the 15th day of treatment, he felt chest throbbing and shortness of breath. During treatment, the patient often complained of tightness in the chest and fever that rose suddenly, so we suspected pneumonia. The patient received antibiotic escalation therapy with meropenem and levofloxacin, but his condition did not improve. Naso/oropharyngeal swab tests were performed 11 times during the course of the treatment, and positive results of SARS-CoV-2 infection were obtained and remained positive, except for the negative results on the 4th and 6th tests. Two consecutive negative results were obtained on the 10th and 11th swabs (Table 1). The patient was uneventfully discharged after 52 days of treatment.

### 2.4 Case 4

**Fig. 4:**
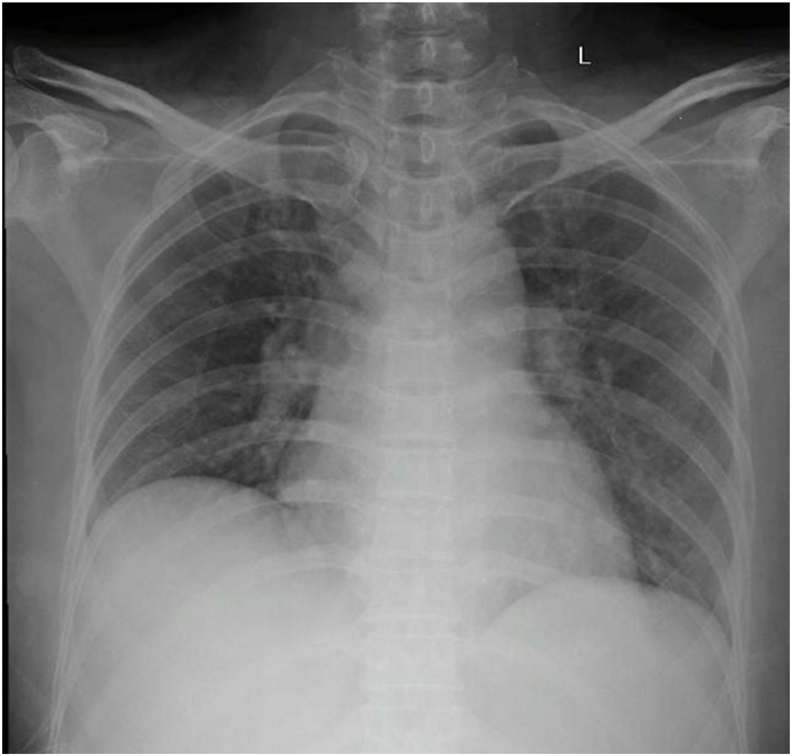
Chest X-ray showed bilateral pneumonia.

A 56-year-old woman came to the emergency department with complaints of lethargy that worsened in the last 6 days before admission. Her complaints included diarrhea more than 10 times daily, without mucus or blood. The patient also experienced fever for 4 days before admission. The patient went to a private hospital, was hospitalized for 4 days, and was referred to our hospital for further tracking related to her neutropenia. She has a history of hyperthyroidism that she had suffered from for the past 1.5 months and has received 100 mg twice daily PTU therapy and thiamazole 5 mg once daily. She had a history of penicillin allergy. Her vital signs were within normal limits. Lung auscultation revealed no apparent abnormality in either lung. Rapid diagnostic tests using SARS-CoV-2 antibody were performed and showed non-reactive results. Chest X-rays showed bilateral pneumonia (Fig. 4), while routine blood tests revealed pancytopenia. On the ninth day of admission, her husband was known to have flu symptoms, had a rapid diagnostic test for SARS-CoV-2 antibodies with reactive results, and had been examined by naso/oropharyngeal swab test with positive results. The patient's husband is known to have a history of contact with people traveling from the local COVID-19 transmission area. After admission, our patient received antibiotics and antiviral therapy based on the COVID-19 Prevention and Control guidelines by the Indonesian Ministry of Health, namely, azithromycin, hydroxychloroquine, umifenovir and lopinavir-ritonavir. Nasal and oropharyngeal swab tests were performed 14 times during the course of the treatment, and positive results of SARS-CoV-2 infection were obtained and remained positive, except for the negative results on the 2nd and 10th tests (Table 1). Two consecutive negative results were obtained on the 13th and 14th swabs (Table 1). The patient was discharged uneventfully after 70 days of treatment.

## 3 Discussion

We report the occurrence of prolonged nucleic acid conversion and the possibility of false negative RT-PCR test results of SARS-Cov-2 from Indonesian patients with COVID-19. To the best of our knowledge, our case is the longest prolonged nucleic acid conversion reported being 70 days. Prolonged nucleic acid conversion is defined as conversion for more than 24 days from the onset of the typical symptoms of COVID-19 [7]. Interestingly, previous studies have reported that symptomatic and asymptomatic patients have the same viral loads, showing that there is a potential for transmission from patients without or with mild symptoms [9,10]. Our cases recovered from mild symptoms of COVID-19 but experienced an extended duration of viral shedding. Moreover, during treatment in the hospital, our cases showed negative results between the positive results of RT-PCR. This finding is similar to previous reports [7,11,12], which reported the high false negative rate of RT-PCR for COVID-19 detection. The fluctuating trend of our RT-PCR results in our cases might be due to insufficient viral material in the specimen, laboratory errors during sampling, restrictions on sample transportation [11], or mutations in the primary and probe target regions in the SARS-CoV-2 genome [13].

RT-PCR is indeed a “gold standard” diagnosis of COVID-19 because it detects RNA but not the active infectious virus. A virus culture study by Bullard et al. [14] reported that virus infectivity decreased significantly when RT-PCR cycle threshold (C_T_) values were >24. C_T_ values of RT-PCR tests are inversely related to viral load. For every 1 unit increase in C_T_, the odds ratio for infectivity decreases by 32%. Some studies also reported that viral infectivity in patients with a symptom duration >8 days may be low [14,15]. Several factors might affect the occurrence of prolonged nucleic acid conversion, including older age, comorbidities, such as diabetes and hypertension, and impaired immune function [16]. Our cases, except patient #1, had comorbidities, such as diabetes, hyperthyroid, asthma and arrhythmia.

Previously, on January 12, 2020, the WHO recommended that COVID-19 patients be discharged from isolation when the patients are clinically recovered and show two negative RT-PCR results on sequential samples taken at least 24 h apart [17]. On May 27, 2020, WHO revised the recommendation for discharging COVID-19 patients from isolation without requiring retesting of RT-PCR: a) for symptomatic patients: 10 days after symptom onset, plus at least 3 additional days without symptoms (including without fever and without respiratory symptoms); b) for asymptomatic cases: 10 days after positive test of RT-PCR for SARS-CoV-2 [18]. Since August 1, 2020, our government has already adopted these recommendations for the management of patients with COVID-19 in Indonesia, except for patients with severe and critical COVID-19: the criteria for discharging patients from isolation needs a negative RT-PCR result [19].

Positive RT-PCR results in COVID-19 patients address the detection of the presence of SARS-CoV-2 RNA, but, this does not essentially mean that a patient is infectious and able to transmit the virus to another person [18]. Several studies have shown that SARS-CoV-2 is unable to be cultured from the respiratory tract swabs of COVID-19 patients after 7–9 days of symptom onset [14,20,21].

The limitation of our study is the small sample size of COVID-19 cases with prolonged nucleic acid conversion and the possibility of false-negative RT-PCR results. These facts should be considered during the interpretation of our case series.

## 4 Conclusions

Our cases further confirmed the occurrence of prolonged nucleic acid conversion and the possibility of false negative RT-PCR results in patients with COVID-19 instead of recurrence of infection.

## Consent

Written informed consent was obtained from the patient for publication of this case series and accompanying images. A copy of the written consent forms is available for review by the Editor-in-Chief of this journal on reasonable request.

## Provenance and peer review

Not commissioned, externally peer reviewed.

## Declaration of competing interest

No potential conflict of interest relevant to this article was reported.
